# Endocytosis and Enamel Formation

**DOI:** 10.3389/fphys.2017.00529

**Published:** 2017-07-31

**Authors:** Cong-Dat Pham, Charles E. Smith, Yuanyuan Hu, Jan C-C. Hu, James P. Simmer, Yong-Hee P. Chun

**Affiliations:** ^1^Department of Periodontics, School of Dentistry, University of Texas Health Science Center at San Antonio San Antonio, TX, United States; ^2^Department of Anatomy and Cell Biology, McGill University Montreal, QC, Canada; ^3^Department of Biologic and Materials Sciences, University of Michigan Ann Arbor, MI, United States; ^4^Department of Cell Systems & Anatomy, School of Medicine, University of Texas Health Science Center at San Antonio San Antonio, TX, United States

**Keywords:** endocytosis, amelogenesis, endocytic trafficking, Rab proteins, clathrin, pinocytosis

## Abstract

Enamel formation requires consecutive stages of development to achieve its characteristic extreme mineral hardness. Mineralization depends on the initial presence then removal of degraded enamel proteins from the matrix via endocytosis. The ameloblast membrane resides at the interface between matrix and cell. Enamel formation is controlled by ameloblasts that produce enamel in stages to build the enamel layer (secretory stage) and to reach final mineralization (maturation stage). Each stage has specific functional requirements for the ameloblasts. Ameloblasts adopt different cell morphologies during each stage. Protein trafficking including the secretion and endocytosis of enamel proteins is a fundamental task in ameloblasts. The sites of internalization of enamel proteins on the ameloblast membrane are specific for every stage. In this review, an overview of endocytosis and trafficking of vesicles in ameloblasts is presented. The pathways for internalization and routing of vesicles are described. Endocytosis is proposed as a mechanism to remove debris of degraded enamel protein and to obtain feedback from the matrix on the status of the maturing enamel.

## Endocytosis in ameloblasts

Enamel formation is a unique process that coordinates the movement of proteins and ions between ameloblasts and the developing extracellular matrix (Smith and Nanci, 1996; Lacruz et al., 2013b). The extracellular matrix represents a sealed compartment between ameloblasts and the mineralized dentin without direct access to the vascular system or the connective tissue compartment (Bronckers, 2016). The transport of proteins and ions between ameloblasts and matrix for crystal mineralization is controlled by ameloblasts. As the enamel organ develops, the inner epithelial cells differentiate into polarized ameloblasts. The two key protein transport functions of ameloblasts are the secretion and the resorption of enamel proteins. Ameloblasts secrete enamel proteins at the surface of forming enamel that assemble into a scaffold to initiate and lengthen the growing mineral crystals (Smith et al., 2016). As enamel proteins are selectively cleaved by proteinases, fragments and perhaps some almost intact proteins are removed from the matrix via endocytosis by ameloblasts, a process that speeds up over time as enamel formation continues (Reith and Cotty, 1967; Smith, 1979; Kallenbach, 1980a,b). The freed up space is then utilized to widen the individual enamel ribbons. The final product contains <5% of proteins and water (Schmitz et al., 2014). The failure of efficient removal of enamel proteins and deposition of mineral results in hypomineralized or hypomature enamel. The enamel proteins constitute the protein backbone of the enamel matrix and include amelogenin, ameloblastin, and enamelin. All of them are part of the cluster called secreted calcium-binding phosphoproteins (Kawasaki et al., 2004). Structurally, all enamel proteins possess a poorly defined secondary structure, a feature characteristic for intrinsically disordered proteins (Wald et al., 2017). Regions of hydrophobic residues of amelogenin facilitate protein-protein interactions resulting in assemblies of nanospheres (Fincham et al., 1995). In the absence of amelogenin, enamel ribbons lose their self-sufficiency and fuse together in fan-like structures (Smith et al., 2016). Enamel proteins are sequentially processed into fragments that are then internalized by ameloblasts (Bartlett, 2013). Initially, matrix metalloproteinase 20 cleaves enamel proteins at highly selective internal sites during the secretory stage (Fukae et al., 1998). The remaining fragments are then further degraded into smaller peptides by kallikrein4 (kallikrein related peptidase 4) during the maturation stage (Nagano et al., 2009).

**Figure 1:**
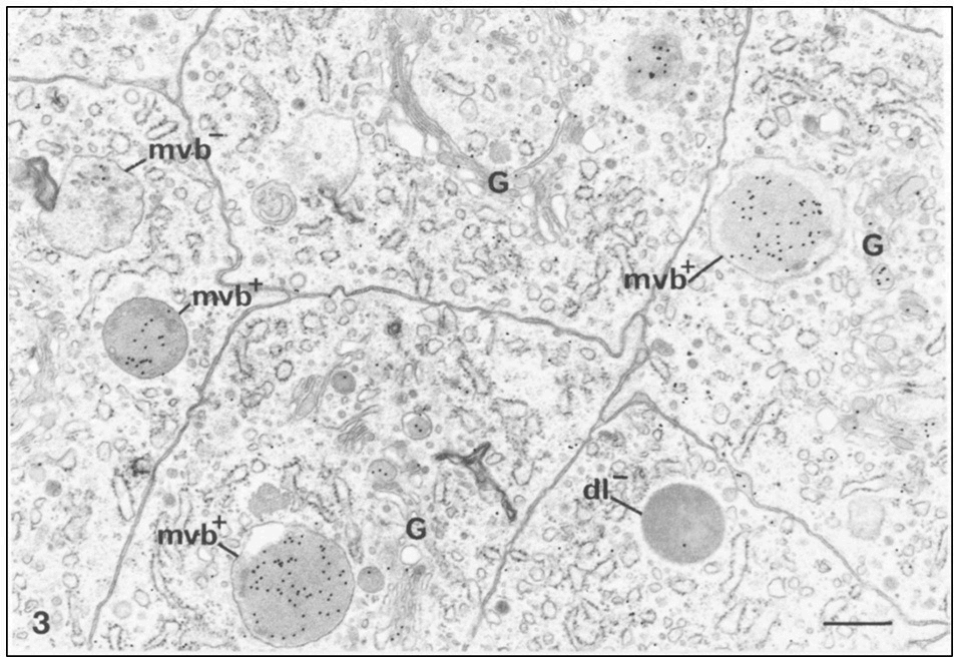
Immunocytochemical preparation illustrating the distribution of gold labeled amelogenin over various compartments of ameloblasts from the secretion stage in mouse incisors. Lysosomes appear variably labeled. Multivesicular bodies are often intensely labeled (mvb+). An unlabeled multivesicular body (mvb−) and an unlabeled dark lysosome (dl−) are shown. The Golgi apparatus (G) shows some labeling by gold particles, × 24,875. Bar = 0.5 μm. Permission to reprint from: Application of High-Resolution Immunocytochemistry To the Study of the Secretory, Resorptive, and Degradative Functions of Ameloblasts by Nanci et al. (1987a).

The uptake of degraded proteins takes place throughout all stages of enamel formation (Ozawa et al., 1983). The endocytic activities of preameloblasts and ameloblasts include the removal of basement membrane proteins and enamel proteins via vesicles and their transport to lysosomes (Katchburian and Holt, 1969; Kallenbach, 1980a; Takano and Ozawa, 1980; Ozawa et al., 1983; Salama et al., 1989, 1990a,b; Smith et al., 1989; Nanci et al., 1996). The localization of amelogenin with immune gold labeling techniques has established that enamel proteins are found in large quantities in organelles with functions in endocytosis in ameloblasts of both secretory and maturation stages (Figure 1, Table 1).

**Table 1:** Density of gold labeling over enamel and organelles in ameloblasts following incubations with anti-amelogenin antibody^a^.

**Compartment**	**Density of labeling**^b^		
	**Early secretion (mouse)**	**Mid secretion (rat)**	**Early maturation (rat)**
Enamel	129.9 ± 9.4	121.4 ± 9.6	80.0 ± 3.9
Rough endoplasmic reticulum	7.2 ± 0.4	8.4 ± 0.4	9.2 ± 0.4
Golgi saccules	17.7 ± 0.8	15.1 ± 1.0	9.8 ± 0.6
Secretory granules	154.7 ± 10.9	137.6 ± 8.3	137.0 ± 14.5
Dark lysosomes	16.1 ± 3.3	8.5 ± 0.7	5.9 ± 0.5
Pale lysosomes	14.8 ± 2.9	11.1 ± 0.9	9.8 ± 1.2
Multivesicular bodies	19.8 ± 3.8	37.0 ± 3.5	26.9 ± 1.3
Mitochondria^c^	3.9 ± 0.7	3.8 ± 0.6	4.1 ± 0.2

*Amelogenin is found in compartments associated with protein synthesis and secretion (rough endoplasmatic reticulum, Golgi, and secretory granules) and endocytosis (dark and pale lysosomes, and multivesicular bodies) pathways*.

a *Data modified from Nanci et al. (1985, 1987b)*.

b *Number of particles / μm^2^ ± SEM*.

c *Index of background labeling*.

*Permission to reprint from: Application of High-Resolution Immunocytochemistry To the Study of the Secretory, Resorptive, and Degradative Functions of Ameloblasts by Nanci et al. (1987a)*.

Endocytosis is linked to enamel mineralization to remove processed enamel proteins from the matrix and to deposit mineral (Smith, 1979, 1998; Nanci et al., 1987a; Smith et al., 1989). The site and configuration of plasma membrane from which enamel proteins are secreted and endocytosed differ in morphology depending on developmental stage. During the pre-secretory stage fragments of the degraded basal lamina are removed through finger-like cell processes penetrating into the pre-dentin (Reith, 1967; Kallenbach, 1976; Nanci et al., 1989). During secretory and maturation stages stippled material adjacent to ameloblasts is interpreted as degraded enamel proteins and their localization is indicative of resorptive activity (Kallenbach, 1980b; Ozawa et al., 1983; Nanci et al., 1987b).

**Figure 2:**
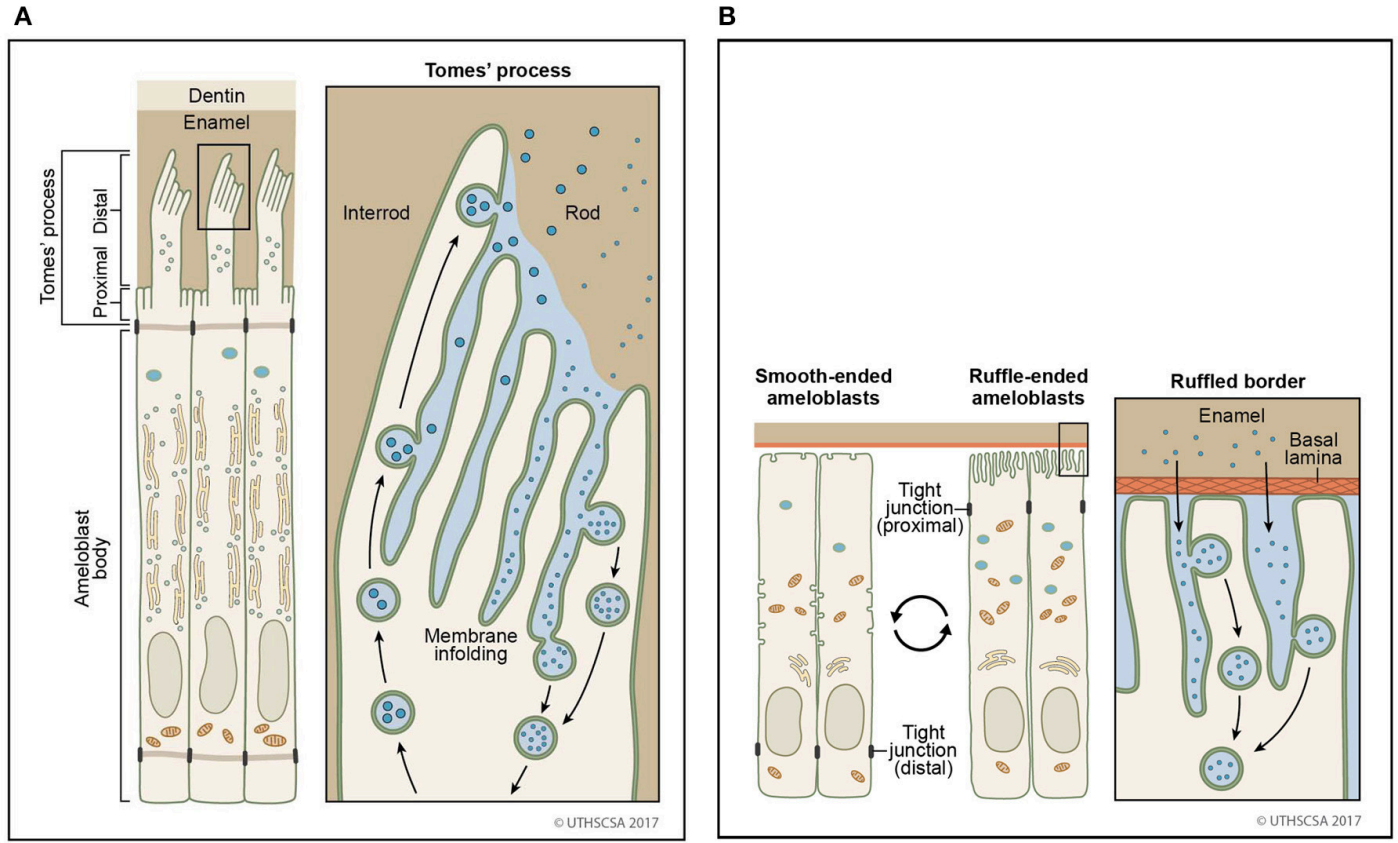
Sites of secretion and endocytosis of the ameloblast membrane. **(A)** The secretory ameloblasts forms a Tomes' process from the apical membrane with a proximal portion and a distal portion. The proximal portion is associated with formation of interrod enamel, the distal portion with rod enamel formation. Vesicle fusion can be observed on the surface membrane adjacent to the rod growth site. Many vesicles fuse (secretion) or originate (endocytosis) from membrane infoldings found on the proximal portion and the distal portion. **(B)** In the maturation stage, degraded enamel proteins are internalized by ameloblasts. Ameloblasts modulate between smooth-ended and ruffle bordered membranes. In 80% of the maturation stage, ameloblasts are ruffle-ended with deep membrane invaginations. Degraded enamel proteins from the enamel matrix permeate the area between convoluted tubules and are resorbed via vesicles.

**Figure 3:**
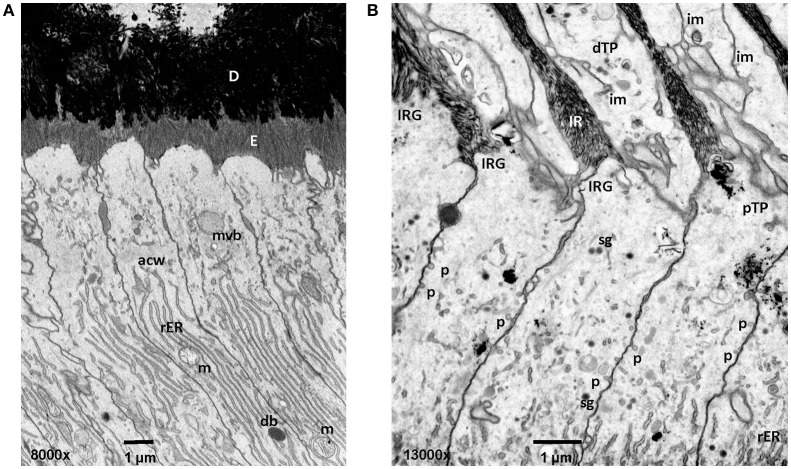
Early and mid secretory stage ameloblasts in mandibular mouse incisors. All procedures involving animals were reviewed and approved by the IACUC committee at the University of Michigan and all relevant guidelines were followed. Handling of animals and tissues was followed according to protocols described earlier (Smith et al., 2016). **(A)** The Tomes' processes are not fully developed in early secretory stage ameloblasts. Enamel crystallite ribbons in the initial interrod layer are oriented perpendicular to dentin and the apical ameloblast membrane. **(B)** In mid secretory ameloblasts pinocytotic vesicles are observed along the non-secreting surface of the Tomes' process and laterally between adjacent ameloblasts below the level of the apical junctional complex. In the distal portion of the Tomes process membrane infoldings form a complex network and are in close relationship to vesicles. D, dentin; E, enamel; mvb, multivesicular body; acw, apical cell web; rER, rough endoplasmic reticulum; m, mitochondria; db, dense body (lysosome); im, membrane infolding; dTP, distal portion of Tomes' process; IRG, interrod growth site; pTP, proximal portion of Tomes' process; sg, secretory granule; p, pinocytotic vesicle.

With the formation of the Tomes' process on the apical membrane during secretory stage, proteins are secreted in large quantities from two growth sites, distal and proximal, to give rise to orientation of rod and interrod enamel (Nanci and Warshawsky, 1984). Both of these sites are characterized by deep membrane infoldings (Weinstock and Leblond, 1971; Kallenbach, 1973; Nanci and Warshawsky, 1984; Uchida and Warshawsky, 1992; Kim et al., 1994). Vesicles with granular content to be secreted are found in close proximity to infoldings suggesting that a membrane fusion event between vesicle and infolding results in the discharge of the luminal content of the vesicle into the channel of the infolding (Simmelink, 1982). The enamel proteins then could escape through the channels between the infoldings to the outer surface of the secretory face (Figures 2, 3). Conversely, it is conceivable that endocytosis could occur in the reverse direction, inside the membrane infolding similar to a tubular network (Smith, 1979). The space inside an infolding ranges from small and narrow to bloated and filled with granular material (Kallenbach, 1974; Nanci et al., 1987b). This mechanism allows several vesicles to fuse in a limited area of the cell surface and to release and internalize material in large quantities. Enamel ribbons are bundled and closely related to the openings of the infoldings and extend in the direction of the opening (Nanci and Warshawsky, 1984). The ameloblast surface seems to be less infolded when there is loss of function of any one of the enamel matrix scaffold proteins (Smith et al., 2016). In the maturation stage, ameloblasts acquire a ruffle-ended membrane 80% of the time compared to smooth-ended borders. Ruffle-ended ameloblasts are more absorptive than smooth-ended ameloblasts (Nanci et al., 1987b). The ruffle-ended membrane forms a complex, infolded apical surface constantly changing its configuration.

In addition to endocytosis of enamel proteins from the apical membranes of secretory and maturation stage ameloblasts, some small amounts of enamel matrix proteins are secreted and endocytosed from the lateral extracellular spaces between the cells (Nanci and Smith, 1992). Granular material containing amelogenin has been found in accumulations between the tight junctions of secretory stage ameloblasts (Nanci and Warshawsky, 1984; Nanci et al., 1987a). These “patches” are associated with sites of the ameloblasts membrane that lack membrane infoldings and mineralization. It was suggested that the microenvironment of rod and interrod growth sites is unique for the initiation of mineralization (Nanci et al., 1987c). At the lateral surfaces pinocytosis is frequently observed (Figure 3).

The packing of vesicles for secretion and endocytosis is a membrane consuming process altering the surface area. Given the large quantity of secreted enamel proteins, the gain of membrane during fusion events could alter the shape of the cell. However, for the Tomes' process maintaining the shape is critical to allow a defined mineralization front. By fusing with the membrane of infoldings or ruffles, membrane is recycled and the outer plasma membrane is not affected. As a result, infoldings and ruffles become longer and branched (Nanci and Smith, 1992).

## Endocytosis types, mechanisms, pathways

Endocytosis is a form of active, energy-using transport of extracellular molecules internalized by a cell into vesicles. As a mechanism to communicate the status of the extracellular environment and the cells, endocytosis is a vehicle for executing cell homeostasis including uptake of nutrients, matrix- cell communication, changes in cell shape and polarity (Mills, 2007; Eaton and Martin-Belmonte, 2014; Villasenor et al., 2016). Beyond the homeostasis of a single cell, endocytosis is essential for the homeostasis of multicellular tissues, organs and communities (Mostov and Cardone, 1995; Mellman, 1996).

The prerequisite for the regulation of endocytosis is that cells are able to receive and respond to external signals (Pelkmans et al., 2005). The interaction can be modulated through different uptake mechanisms and through ligand-receptor binding, inducing specific cellular functions (Le Roy and Wrana, 2005). Identified uptake pathways include non-specific macropinocytosis and specific receptor-mediated/clathrin-mediated and caveolae/raft-mediated endocytosis (Racoosin and Swanson, 1992; Swanson and Watts, 1995; Mellman, 1996; Conner and Schmid, 2003). The interaction between receptor and ligand triggers a change in the conformation in the cytosolic tail that includes motifs for internalization (Dahlen et al., 2003; Pandey, 2009). These motifs are tyrosine or dileucine based and are critical for internalization efficiency and for routing the cargo to the intended designation (Collawn et al., 1993; Dahlen et al., 2003; Pandey, 2009).

### Clathrin-mediated endocytosis

The most extensively described endocytosis pathway is clathrin-mediated endocytosis. The generation of coated vesicles was discovered in mosquito oocytes (Roth and Porter, 1964). Each clathrin subunit consists of three large (heavy) and three small (light) polypeptide chains resembling a triskelion, a three-legged structure (Pearse, 1975, 1976). Clathrin molecules self-assemble into a 3-dimensional lattice supported by the heavy chains in the shape of a basket. The assembly and disassembly of clathrin around the vesicle is controlled by the clathrin light chains (Pearse, 1976). Clathrin uses adapter proteins (AP) to bind to membranes or cargo (Pearse et al., 2000; Sorkin, 2004). Clathrin-mediated endocytosis regulates the internalization and recycling of receptors employed in cellular activities. Some examples are signal transduction, cell adhesion, cell proliferation, nutrient uptake and synaptic vesicle reformation (Polo and Di Fiore, 2006; Saheki and De Camilli, 2012; Antonescu et al., 2014).

Vesicles found in preameloblasts, Tomes' processes of secretory ameloblasts and maturation stage ameloblasts are either coated or non-coated (Smith, 1979; Ozawa et al., 1983; Sasaki, 1984a,b; Franklin et al., 1991; Uchida and Warshawsky, 1992). Compared to uncoated vesicles originating either from secretion or endocytosis, coated vesicles are inactive for the internalized vesicles via the clathrin-mediated pathway. Coated vesicles are described in electron micrographs of secretory stage ameloblasts with a size of 0.1–0.12 μm in diameter (Reith and Cotty, 1967; Garant and Nalbandian, 1968). In presecretory ameloblasts, coated vesicles are found in the cytoplasmic protrusion that penetrate the basal lamina and facilitate its degradation (Katchburian and Burgess, 1983). In secretory stage ameloblasts, extracellular material is internalized by coated vesicles and tubulovesicular structures (Sasaki, 1984a). Coated vesicles have a tight relationship to tubules residing in the core of the Tomes' process (Uchida and Warshawsky, 1992). Tubules branch out from the core and are part of a network. In maturation stage ameloblasts, coated vesicles are found in ruffle-ended ameloblasts that are filled with fine granular material (Sasaki, 1984b). In contrast, smooth-ended ameloblasts contain only few coated vesicles (Kallenbach, 1980a; Sasaki, 1984b). The morphological difference in the apical membrane of maturation stage ameloblasts suggests that their function is dedicated to different processes with high resorptive activities associated with a ruffled border and low resorptive, but homeostasis activities with a smooth-ended border (Figure 2; Takano and Ozawa, 1980).

The endocytosis of amelogenin in enamel organ epithelium is proposed via clathrin dependent endocytosis involving the receptor proteins lysosome-associated membrane protein 1 (Lamp1) and cluster of differentiation 63 (CD63) (Shapiro et al., 2007; Lacruz et al., 2013a). Lamp1 and CD63 are transmembrane proteins routed between the membranes of the cell surface and lysosomes via endocytosis and via recycling pathways. Lamp1 and CD63 may interact with AP-2 clathrin AP for the uptake of clathrin-coated vesicles in ameloblasts (Lacruz et al., 2013a). The regulation of endocytosis may involve the microRNA miR-153 through interactions with Lamp1 and clathrin (Yin et al., 2017).

### Pinocytosis

Fluid-phase endocytosis called pinocytosis can be distinguished by the size of the pinosomes as macropinocytosis and micropinocytosis. While macropinocytosis marks the uptake of fluid phase, micropinocytosis is associated with receptor-mediated and fluid-phase uptake.

Fluid phase endocytosis represents an uptake mechanism documented as occurring in ameloblasts. The process of fluid phase endocytosis trafficking of cargo can be demonstrated by supplying exogenously provided horseradish peroxidase (HRP) and observing the intracellular localization of HRP in cell organelles to which HRP is transported. For this technique, HRP is intravenously injected as a 5% solution and animals are sacrificed after 15–90 min (Sasaki, 1984c). During secretory stage, the apical terminal bars are not permeable for HRP (Kallenbach, 1980b). HRP accumulates at rod and interrod growth sites. The uptake of large quantities of HRP takes place at the Tomes' process and is subsequently trafficked to endosomes and lysosomes (Kallenbach, 1980b; Matsuo et al., 1986). In maturation stage ameloblasts, the apical and basal tight junctions open and close as ameloblasts modulate between ruffle-ended and smooth-ended forms and allow HRP to reach intercellular spaces between ameloblasts and close to the papillary layer (Sasaki et al., 1983a). HRP is rapidly internalized in large quantities and is carried forward in coated pits, vesicles, multivesicular bodies (MVB) and tubulovesicular structures (Sasaki and Higashi, 1983; Sasaki et al., 1983b; Sasaki, 1984a,c). Accumulated HRP is incorporated from the cell membrane into the cytoplasm through pinosomes and pinocytotic coated vesicles (Sasaki, 1984a). The pinosomes then fuse to form large endocytic vesicles. HRP is accumulated in the endocytic vacuoles and MVB which serve as a carrier for HRP (Sasaki, 1984c). High magnification focused ion beam micrographs reveal pinocytotic activity at the lateral membranes of the proximal portion of the Tomes' Process (Figure 3). HRP can be followed from internalization through the endocytic compartment to the lysosomes where it is digested by lysosomes (Kallenbach, 1980b).

### Phagocytosis

Phagocytosis describes the uptake of a solid particle (>0.5 μm) by the cell to form an internal compartment known as a phagosome (Gordon, 2016). In unicellular eukaryotes, phagocytosis serves in the acquisition of nutrients. In mammalian cells, phagocytosis is a mechanism for immune cells, such as macrophages, neutrophils, and dendritic cells to remove pathogens, damaged cell organelles and dead cells. In contrast to pinocytosis endosomes, phagosomes can be as large as the phagocyte, depending on the size of the ingested particle. Phagocytosis is initiated by membrane protrusions (filopodia) in direction of the particle and through binding of the particle to cell surface receptors. Pathogen-associated molecular patterns, Fc regions of antibodies, complement molecules and apoptotic cells are recognized by cell surface receptors (Flannagan et al., 2012; Gordon, 2016). After ingestion of the particle the phagosome fuses with a lysosome where the particle is exposed to degradation and microbicidal action.

After completion of the secretory stage, ameloblasts go through a brief shift to enter the maturation stage. This shift is accompanied by notable changes in ameloblasts morphology from tall polarized cells with a Tomes' process to shorter polarized cells without an apical process. In rodent incisors, this change occurs within 19 hours (Smith and Warshawsky, 1977). About 25% of transitional stage ameloblasts perish into cellular debris of varying size found between and below ameloblasts (Moe and Jessen, 1972). Cellular debris is distinct from ameloblasts with their dense cytoplasm surrounded by wide intercellular spaces between ameloblasts. It can cross intercellular spaces to the adjacent cell layer (stratum intermedium). Cells of the stratum intermedium form cytoplasmic processes to engulf ameloblast debris (Moe and Jessen, 1972). During the transition and maturation stages, macrophages are present in the forming papillary layer that are involved in the removal of cellular debris (Jessen and Moe, 1972; Nishikawa and Sasaki, 1996).

## Endocytic trafficking of vesicles

Vesicles that originated by phagocytosis or pinocytosis contain cargo in the form of bound ligands or extracellular liquid phase material. The movement of vesicles within the cell to their destination compartment or organelle is called endocytic trafficking. Newly formed vesicles are first transported into early endosomes to sort them based on their content and to direct them to their destination. Endosomes can progress to lysosomes to degrade their content or fuse with the plasma membrane to return receptors and release the content to the environment. A group of proteins in the Ras superfamily of GTPases (Rab proteins) have been identified as the molecular machinery that regulates membrane trafficking pathways (Segev, 2001). They take part in vesicle formation, motility, docking, membrane remodeling and fusion (Segev, 2001).

### Ras superfamily of GTPases

**Figure 4:**
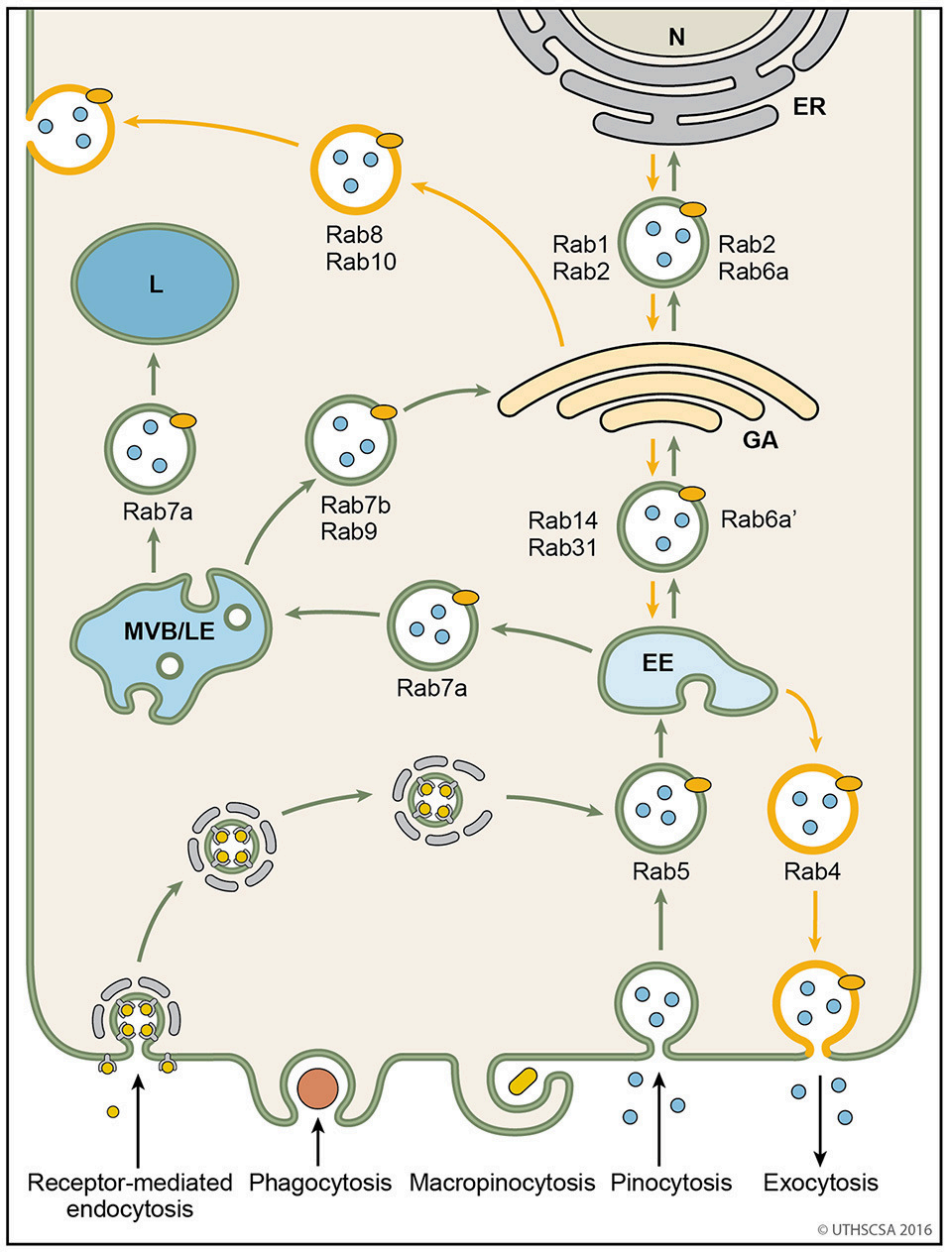
Vesicular trafficking pathways during endocytosis. Schematic diagram showing the general pathways of internalized material. Molecules are taken up into vesicles via various types of endocytosis (receptor-mediated, phagocytosis, micropinocytosis, pinocytosis). Vesicles (coated and non-coated) fuse with EE, the first cellular sorting station, where the cargo is distributed to MVB/LE, GA or back to the plasma membrane (recycling pathway) via their respective Rab proteins. Cargo which is destined for degradation in lysosomes, is transported from EE to lysosomes via MVB/LE. At MVB/LE vesicles can still enter the recycling pathway via GA. Decreasing pH in the EE, MVB/LE, and lysosome are indicated by different shades of blue. EE, early endosome; ER, endoplasmatic reticulum; GA, Golgi apparatus; L, lysosome; MVB/LE, multivesicular body/late endosome; N, nucleus.

Rab proteins constitute the largest branch of the Ras superfamily with over 70 members. They are small GTPases/GTP-binding proteins of 21–25 kDa and localize to the cytosolic periphery of the membrane. As the predominant regulators of trafficking of endocytic vesicles, they control the un-coating, tethering, and membrane fusion and are executed by different types of Rabs (Hutagalung and Novick, 2011; Jena, 2011). The directionality of the vesicles is determined by the type of Rab protein localizing with the membrane of the endocytic organelle (Table 2, Figure 4; Hutagalung and Novick, 2011). Rab genes are highly conserved from yeast to human (Colicelli, 2004). Rab GTPases interact with the cytosolic aspect of the intracellular compartment to regulate and direct vesicles along different pathways. Through their effectors, Rab GTPases control vesicle formation, vesicle movement mediated by microfilaments, and membrane fusion (Hammer and Wu, 2002; Short et al., 2002).

**Table 2:** Intracellular proteins in endocytic, transcytic, and exocytic pathways.

**Protein**	**Human genes**	**Intracellular localization**	**Function**	**References (cloning, localization, function)**
**RAB PROTEINS**				
Rab1	*RAB1A, RAB1, YPT1*	ER–Golgi intermediate	Anterograde trafficking from ER to Golgi	Zahraoui et al., 1989; Tisdale et al., 1992; Zerial and McBride, 2001
Rab2	*RAB2A, LHX, RAB2*	ER–Golgi intermediate	Retrograde trafficking from Golgi to ER	Zahraoui et al., 1989; Chavrier et al., 1990; Tisdale et al., 1992
Rab4	*RAB4A*	EE and RE	Trafficking from EE and RE to plasma membrane	Zahraoui et al., 1989; van der Sluijs et al., 1992; Seachrist and Ferguson, 2003
Rab5	*RAB5A, RAB5*	Clathrin coated vesicles and EE	Endocytic internalization and EE fusion	Zahraoui et al., 1989; Chavrier et al., 1990; Somsel Rodman and Wandinger-Ness, 2000
Rab7a	*RAB7A, PRO2706*	LE	Trafficking from EE to LE and from LE to lysosomes	Chavrier et al., 1990; Harrison et al., 2003; Guerra and Bucci, 2016
Rab7b	*RAB7B, RAB7*	LE	Trafficking from LE to TGN	Surmacz et al., 2006; Progida et al., 2010
Rab8	*RAB8A, MEL, RAB8*	Median Golgi and TGN	Trafficking from median Golgi and TGN to basolateral membrane	Huber et al., 1993; Chen and Wandinger-Ness, 2001; Peranen, 2011
Rab9	*RAB9A, RAB9*	LE	Retrograde transport from LE to trans-Golgi	Lombardi et al., 1993; Soldati et al., 1995; Davies et al., 1997
Rab10	*RAB10*	Golgi	Trafficking and recycling from Golgi to basolateral membrane	Chen et al., 1993; Bao et al., 1998; Schuck et al., 2007
Rab14	*RAB14, FBP*	EE and Golgi	Transport from Golgi to EE	Elferink et al., 1992; Junutula et al., 2004; Proikas-Cezanne et al., 2006
Rab31 (alternate name Rab22b)	*RAB31*	EE and TGN	Anterograde transport from TGN to early endosomes	Rodriguez-Gabin et al., 2001; Ng et al., 2007

*ER, endoplasmatic reticulum; EE, early endosome; RE, recycling endosome; LE, late endosome; TGN, trans-Golgi network*.

In the enamel organ, a limited number of Rab GTPases have been investigated. Rab10 and Rab24 were localized to ameloblasts of the maturation stages and papillary cells (Lacruz et al., 2013a). Rab10 assists in the trafficking of vesicles from the Golgi apparatus to the basolateral membrane (Schuck et al., 2007). Rab24 is found in the endoplasmatic reticulum, cis-Golgi and late endosomes related to autophagy (Munafo and Colombo, 2002). In presecretory to secretory ameloblasts Rab23 was localized possibly negatively regulating sonic hedgehog signaling (Miletich et al., 2005).

### Endosomal vesicles

Endosomes are membrane-bound compartments inside of eukaryotic cells containing material that was internalized from the exterior of the cell. Their function is to sort and transport vesicles containing internalized solutes, receptors, lipids or pathogenic agents. Through their cargo, they also carry information from the extracellular compartment into the cell critical for cell morphology, maintenance and response to signals (Villasenor et al., 2016). Efficient sorting routes the vesicles to their destinations within the cells, such as the Golgi apparatus, lysosomes and plasma membrane. The two major sorting stations are the early and late endosomes (Mellman, 1996; Russell et al., 2006). Among the early endosomes, late endosomes, and lysosomes exists a dynamic and adaptable continuum with transient hybrid forms. An endosome fused with a lysosome is called an endo-lysosome. The endocytosis pathway is versatile because the organelles undergo continuous maturation, transformation, fusion, and fission (Huotari and Helenius, 2011). The following paragraphs describe the endocytic pathways of vesicles following internalization from the extracellular matrix (Figure 4).

#### Early endosomes

Early endosomes are the initial endocytic vesicle to accept incoming internalized molecules (Gruenberg et al., 1989). Their shapes vary from thin tubes (~60 nm diameter) to large spheres (~400 nm diameter). As membrane invaginations and scission events occur simultaneously on the endosome membrane, the shape of the endosomes underlies dynamic changes (Gruenberg, 2001). Proteins targeted for recycling may accumulate within tubular membranes. Early endosomes are a hub for sending vesicles off to late endosomes, recycling endosomes, vesicles from the Trans Golgi network or lysosomes. Internalized vesicles are assisted in their transport to and fusion with early endosomes by Rab5. Rab5 tethers to the membranes of cells and endosomes via a C-terminal hydrophobic isoprenoid moiety (Peter et al., 1992; Desnoyers et al., 1996; Shen and Seabra, 1996). In addition to controlling the origination of vesicles from the plasma membrane, vesicle Rab5 helps to recruit Rab7 as endosomes progress from early to late endosomes via fusion events (Gorvel et al., 1991; Huotari and Helenius, 2011). The fusion of endosomes is facilitated by effector proteins including early endosome antigen 1 (EEA1), rabenosyn5 and multiprotein complex C core vacuole/endosome tethering (CORVET) (Rubino et al., 2000; Balderhaar and Ungermann, 2013; Gautreau et al., 2014).

#### Trans-golgi network and recycling endosomes

The trans-Golgi network (TGN) is the site for sorting newly translated membrane and secretory proteins (Griffiths and Simons, 1986; Mellman and Warren, 2000). Several Rab proteins carry out the vesicle transport from the TGN to early endosomes or plasma membrane for release (Figure 4). Rab31 transports vesicles bidirectionally between the TGN and early endosomes (Rodriguez-Gabin et al., 2009). Vesicles with Rab1 and Rab2 are transported from the TGN to the endoplasmatic reticulum and vice versa, respectively (Plutner et al., 1991; Tisdale et al., 1992, 2004). Rab8 is recruited for the transport of vesicles destined to release proteins from the TGN to basolateral membranes (Huber et al., 1993). Vesicles arriving at the TGN from either the endoplasmatic reticulum or the late endosome are bound for secretion at the plasma membrane via Rab4 (van der Sluijs et al., 1992; Junutula et al., 2004).

Synthesis and secretion are major functions of ameloblasts during enamel formation. The morphology of ameloblasts changes greatly when they differentiate and begin appositional growth of the enamel layer thereby initiating the secretory stage. Secretory stage ameloblasts in rodent incisors, for example, have width to height dimensions of approximately 4–60 μm (1:15). The extreme extension of the ameloblasts facilitates the accommodation of a large number of pronounced cell organelles to synthesize and secrete enamel proteins and to support enamel mineralization. After transcription into mRNA in the nucleus and protein translation in the rough endoplasmatic reticulum, the synthesized enamel proteins progress through the centrally located and very large tubular-shaped Golgi apparatus for post-translational modification and packaging into secretory granules. The supranuclear cytoplasm between nucleus and the Tomes' process is filled with rough endoplasmatic reticulum, Golgi apparatus cisternae, and vesicles, forming an intricate network of membranes in constant exchange of membrane and cargo (Garant and Nalbandian, 1968; Warshawsky, 1968; Sasaki, 1983a; Sasaki and Higashi, 1983). The Golgi cisternae are oriented perpendicular to the nucleus, following the long axis of the cell body. They occupy much of the supranuclear compartment between the nucleus and the microfilaments of the apical cell web with the dimension of 25 × 1.5 μm, but do not penetrate into the Tomes' process (Kallenbach et al., 1963; Garant and Nalbandian, 1968). The saccular stacks of the Golgi apparatus adopt an open-ended, tubular structure and are polarized with shorter saccular stacks on the periphery and long flattened cisternae internally located (Garant and Nalbandian, 1968; Nanci et al., 1993). The endoplasmatic reticulum surrounds the Golgi apparatus in the periphery.

The number of vesicles shuttling between the rough endoplasmatic reticulum, the Golgi apparatus and the plasma membrane is large (Garant and Nalbandian, 1968). They accumulate in the Tomes' process close to the secretory face (Reith, 1967; Garant and Nalbandian, 1968). Vesicles with a dense content are, in average, of 0.8–0.16 μm in diameter (Garant and Nalbandian, 1968; Warshawsky, 1968) and contain enamel proteins to be released at the plasma membrane (Uchida et al., 1991).

All secretory stage enamel proteins, amelogenin (Nanci et al., 1987a, 1989; Inage et al., 1989), enamelin (Uchida et al., 1991; Dohi et al., 1998), and ameloblastin (Lee et al., 1996; Murakami et al., 1997; Uchida et al., 1998) were found in the Golgi apparatus of ameloblasts. They have also been identified in secretory vesicles and at the secretory face of the Tomes' process (Nanci et al., 1989; Uchida et al., 1991). One of the commonalities of the enamel proteins as part of the secreted calcium-binding phosphoprotein cluster is that all members are phosphorylated on a conserved serine residue in a SXE motif of exon 3 (Kawasaki et al., 2004). The enzyme catalyzing the phosphorylation has been identified as Golgi-localized casein kinase, encoded by FAM20C (Tagliabracci et al., 2012), localized to the Golgi apparatus of ameloblasts (Wang et al., 2013). The phosphorylation of enamel proteins is essential as mutations lead to non-lethal Raine syndrome with hypoplastic amelogenesis imperfecta (Acevedo et al., 2015).

After the completion of the secretory stage, maturation stage ameloblasts reduced their height, and the Golgi apparatus reduces its dimensions (Nanci et al., 1993). Most of the enamel proteins synthesized during the secretory stage are greatly reduced in expression by maturation stage ameloblasts (Somogyi-Ganss et al., 2012). They start to produce and secrete the basement membrane proteins amelotin (Holcroft and Ganss, 2011) and ODAM (Iwasaki et al., 2005; Nishio et al., 2010; Dos Santos Neves et al., 2012), implicated in the mineralization of the enamel surface (Abbarin et al., 2015; Nakayama et al., 2015; Núñez et al., 2016).

#### Late endosomes

Along the endocytic pathway, early endosomes mature into late endosomes (Huotari and Helenius, 2011). Late endosomes are a sorting center to direct cargo to the lysosome, Golgi apparatus or opposite plasma membrane (recycling, transcytosis). In the course of conversion from early to late endosomes, Rab5 is removed and replaced with Rab7 (Rink et al., 2005) which is controlled by the membrane protein complex SAND-1/Mon-1 and HOPS (homotypic fusion and protein sorting) (Rink et al., 2005; Peralta et al., 2010; Poteryaev et al., 2010; Plemel et al., 2011). The late endosome is recruited to the target lysosome and tethered onto the lysosomal membrane by HOPS accomplishing the fusion (Balderhaar and Ungermann, 2013). Late endosomes provide an intersection for arriving and leaving vesicles and have a size between 250 and 1,000 nm (Huotari and Helenius, 2011). Incoming vesicles bring information about the environmental conditions and nutrients for the cell. Outgoing vesicles can carry signals for protein synthesis, secretion, endocytosis, recycling, and autophagy. Fusion events with other endosomes and lysosomes are executed by Rab7. Once the endosome fuses with a lysosome, the vesicle content will be degraded and the endosomal pathway cannot be re-entered (Luzio et al., 2007). Endocytic vesicles contain acid hydrolases that operate in an acidic environment and are indicative for degradation (Yamashiro and Maxfield, 1987).

Molecular markers for late endosomes in ameloblasts have not been described. In electron microscopy images unique features of late endosomes can be identified in ameloblasts in secretory and maturation stages with intraluminal vesicles (Katchburian et al., 1967; Reith and Cotty, 1967; Garant and Nalbandian, 1968; Katchburian and Holt, 1969; Smith, 1979; Matsuo et al., 1986; Nanci et al., 1987a, 1993; Salama et al., 1989; Franklin et al., 1991; Uchida and Warshawsky, 1992). MVB originate from fusion with early endosomes and lysosomes and can release their content into the extracellular environment. They represent a type of late endosomes and are found during the entire life cycle of ameloblasts. MVB are first found in pre-secretory ameloblast associated with finger-like projections and the disruption of the basement membrane between odontoblasts and pre-ameloblasts (Reith, 1967; Nanci et al., 1989). In ameloblasts of the secretory stage, MVB reside in the supranuclear zone of the cell (Sasaki, 1983b). During the maturation stage, the resorptive activity at the ruffled border is intense. Large quantities of HRP labeled fluid-phase material are resorbed via pinosomes and delivered to MVB (Sasaki, 1984c). The number of MVB is greater in early maturation compared to late maturation stage (Salama et al., 1990a).

Multivesicular bodies (MVB) containing amelogenin protein have been found with immune gold labeling techniques in secretory-stage, smooth-ended, and ruffle-ended ameloblasts (Nanci et al., 1987a, 1993). However, immunolocalization studies do not answer the question whether the proteins had intracellular or extracellular origin.

#### Lysosomes

Lysosomes originate from late endosomes. The transition between these two organelles is continuous, often forming an endo-lysosome before it transforms into a secondary or dense lysosome (Luzio et al., 2007; Huotari and Helenius, 2011). Lysosomes are the cell's primary degradation center and the terminal station in the endocytic pathway. They contain the breakdown of proteins, polysaccharides, and lipids catalyzed by a wide array of lipases, proteases, and glycosidases in an acidic environment (Luzio et al., 2007; Huotari and Helenius, 2011; Xu and Ren, 2015). Lysosomes generate an acidic luminal pH to activate hydrolytic enzymes and degrade. Acidification is accomplished by proton transport into the lysosome lumen countered by chloride (Dell'Antone, 1979; Ohkuma et al., 1982; Nelson et al., 2000; Sun-Wada et al., 2004; Mindell, 2012). Among lysosomal hydrolases, cathepsins play a major role in peptide degradation. Cathepsins are classified into serine, aspartic and cysteine proteases referring to the amino acid residue in the catalytic center. Cathepsins A (also called human protective protein) and G contain a serine residue in their active site (Kawamura et al., 1980; Rudenko et al., 1995; Hof et al., 1996), while cathepsins D and E have an aspartic acid residue in their active site (Shewale and Tang, 1984; Ostermann et al., 2004). Accordingly, cathepsins B, C (also known as dipeptidyl peptidase I), F, H, K, L, O, S, V, W, and X each feature a cysteine residue in the catalytic site (Turk et al., 2012). While cathepins B, H, L, C, X, F, O, and V are ubiquitously expressed (Turk et al., 2012), cathepsins K, W, and S are only found in specific tissues. Cathepsin K, for example is highly expressed in osteoclasts, and in many epithelial cells (Drake et al., 1996; Buhling et al., 1999; Salminen-Mankonen et al., 2007).

In enamel formation lysosomes are found in ameloblasts at presecretory, secretory and maturation stages (Katchburian and Burgess, 1983; Nanci et al., 1987a,b). Lysosomal activity for acid phosphatase has been demonstrated in preameloblasts, secretory and maturation stage ameloblasts located in granules within the center of the supranuclear region and in the Tomes' process (Katchburian et al., 1967). In preameloblasts, the target of the resorptive function is the basal lamina facing odontoblasts (Katchburian and Burgess, 1983; Uchida et al., 1989). During secretory and maturation stages, enamel proteins are removed from the matrix through resorption by ameloblasts (Reith and Cotty, 1967; Nanci et al., 1987a,b; Salama et al., 1990a). Large lysosomes can have smooth or rough surfaces in maturation stage ameloblasts (Nanci et al., 1993). The shape of lysosomes is described as spherical and elongated, tubular with sizes ranging from 80 to 140 nm in length (Salama et al., 1989).

Lysosomal enzymes present in ameloblasts include acid phosphatase, β-glucoronidase, and leucyl-naphthylamidase (Beynon, 1972). The distinction between pale and dark lysosomes was derived from the electron density of deposits observed with electron microscopy. Pale lysosomes stain inconsistently with inorganic trimetaphosphatase and acid phosphatase. In contrast, dark lysosomes are reliably positive for inorganic trimetaphosphatase (Nanci et al., 1987b). Dark lysosomes contain less protein than pale lysosomes as they are secondary lysosomes with ongoing protein degradation (Nanci et al., 1987a). Ruffle-ended ameloblasts show more endocytic activity than smooth-ended ameloblasts (Nanci et al., 1987b). Interestingly, the lysosomal activity of ameloblasts is higher during secretory stage compared to maturation stages (Table 1). Among the enamel proteins, amelogenin localizes to lysosomes (Nanci et al., 1987b; Inage et al., 1989). Whether enamelin localizes to lysosomes is not clear since the antibody was raised against a protein species of 50–70 kDa potentially containing enamelin and/or ameloblastin (Inage et al., 1989). Much of the degradative effort in lysosomes is associated with cathepsin B, D, F, H, K, L, O, S, and Z expressed by maturation stage ameloblasts (Tye et al., 2009).

## Perspectives and future directions

Enamel mineralizes and matures as a result of a balance between synthesis, deposition, degradation and internalization. The homeostasis between ameloblasts and matrix is critical for proper enamel formation. The intracellular transport of vesicles is important for polarization of epithelial cells, serving as a mechanism to regulate differentiation (Bryant and Mostov, 2008; Butler and Wallingford, 2017). Endocytosis is a fundamental cell function executed during all stages of the ameloblast life cycle to create the most mineralized hard tissue. Since the enamel matrix represents a secluded compartment surrounded by dentin and ameloblasts (Bronckers, 2016), ameloblasts are granted full control of enamel formation. No other cell type has direct access to the enamel matrix. As a result, ameloblasts closely monitor the transport of ions and proteins across the membrane, necessary to control crystal growth. Endocytosis may be a mechanism for communicating changes in the maturing enamel to the ameloblasts. Each of the various stages of amelogenesis has specific criteria that need to be fulfilled to allow cells to advance to the next stage. Disruption of the completion of a stage results in severe cell pathology as caused by absence of any of the enamel proteins (amelogenin, ameloblastin, enamelin), matrix metalloproteinase 20 or kallikrein4 (Fukumoto et al., 2004; Simmer et al., 2009; Bartlett et al., 2011; Hu et al., 2014, 2016). The inability to cleave the enamel proteins is associated with hypomineralized enamel and matrix-retained proteins (Simmer et al., 2009; Bartlett et al., 2011), allowing the conclusion that cleavage and degradation of enamel proteins are a prerequisite for effective endocytosis. Appropriate feedback to ameloblasts is required to maintain ameloblast function (Chun et al., 2010).

Aside from releasing and internalizing vesicle content, the vesicle membrane may supply or reduce the plasma membrane surface when vesicles fuse with the membrane or are pinched off from the membrane. Thereby, the shape and size of the cell may be modified. During the life cycle of an ameloblast, the cell undergoes significant changes in cell shape. During the development of the Tomes' process in preameloblasts, the membrane surface is enlarged which could be supplied by secretory vesicles. During this event, secretion may dominate over endocytosis. Once the Tomes process has been established in secretory stage ameloblasts, secretion and endocytosis may take place in equilibrium. Given the intense protein synthesis activity of secretory ameloblasts, a mechanism is needed to attain homeostasis of membrane surface and the overall cell shape. The three-dimensional shape of the Tomes' process coordinates enamel ribbons in the characteristic rod and interrod enamel. The forming interrod enamel completely surrounds the protruded distal portion of the Tomes' process like prongs. The interrod enamel originates from the proximal portions of the Tomes' process. If ameloblasts were separated from the forming enamel, a cavity in which the Tomes' process resides would become visible from a baso-apical direction. The interrod enamel would be elevated and form a rim surrounding a cavity in the shape of the Tomes' process. The interrod is partially mineralized, and therefore may restrict the size and shape the Tomes' process is able to adopt. When a secretory vesicle undergoes fusion with the surface membrane of a cell, the vesicle experiences a gain in membrane and increases the overall surface of the cell. For the Tomes' process, a gain in membrane from secretory vesicles would alter and enlarge the shape of the Tomes' process. However, being encased by rigid rod and interrod enamel, an enlarged Tomes' process would collide with enamel ribbons. Endocytosis, on the other hand, removes plasma membrane from the cell surface when vesicles are pinched off and trim the contour of the Tomes' process. Invaginations of the plasma membrane of the Tomes' process present an elegant solution to offer surface membrane, but because it is shifted internally, variability in the shape due to membrane fusion from secretory or endocytic vesicles is bypassed. As a result, the surface membrane and the shape of the Tomes' process remain largely unaltered. When the ameloblast progresses from secretory stage to transition stage and maturation stages, the cell's dimensions are reduced and the Tomes' process is dismantled. A preference of endocytosis events would facilitate a reduction in cell surface membrane. Ruffle-ended maturation stage ameloblasts display high activity in endocytosis. Throughout amelogenesis, secretion and endocytosis are highly prominent operations in ameloblasts that may participate in the control of the cell membrane surface available to form and disassemble cell appendages and infoldings and invaginations.

During their life cycle, ameloblasts adopt many different morphologically distinct appearances as preameloblast, secretory ameloblast, transition stage ameloblast, ruffle-ended ameloblast, smooth-ended ameloblast, and reduced enamel epithelium. The sites of endocytosis on the ameloblast membrane are different in each stage. Small processes are protruded into the basal lamina in preameloblasts. The Tomes' process of the secretory ameloblast is a very large projection from the apical membrane. The infoldings of the membrane distal portion form inwards in the Tomes' process. The ruffled border of maturation stage ameloblasts is formed much differently from the secretory stage infoldings associated with rod and interrod growth sites. They sink into the supranuclear cytoplasmic mass. The stage-specific mechanisms and pathways of endocytosis in ameloblasts are not well defined. While strong evidence is available to document vesicles generated during endocytosis, so far, only clathrin-mediated endocytosis and pinocytosis have been identified in ameloblasts as internalization routes. Lipid rafts or caveolae-mediated endocytosis as a clathrin independent form of endocytosis has not been described in ameloblasts. Future directions in understanding endocytosis in ameloblasts should address the feedback mechanisms, differences in endocytosis types depending on the vesicle origin from the infolding of the Tomes' process vs. ruffled border vs. smooth-ended border vs. lateral membrane and the trafficking of endosomes.

## Author contributions

CP and YC drafted the manuscript with help from CS. JH, JS, and CS planned experiments for Figure 3. YH and CS performed experiments for Figure 3. All authors contributed to the manuscript and approved it.

### Conflict of interest statement

The authors declare that the research was conducted in the absence of any commercial or financial relationships that could be construed as a potential conflict of interest.
